# Dimerization of inositol monophosphatase *Mycobacterium tuberculosis *SuhB is not constitutive, but induced by binding of the activator Mg^2+^

**DOI:** 10.1186/1472-6807-7-55

**Published:** 2007-08-28

**Authors:** Alistair K Brown, Guoyu Meng, Hemza Ghadbane, David J Scott, Lynn G Dover, Jérôme Nigou, Gurdyal S Besra, Klaus Fütterer

**Affiliations:** 1 School of Biosciences, The University of Birmingham, Edgbaston, Birmingham B15 2TT, UK; 2 National Centre for Macromolecular Hydrodynamics, School of Biosciences, University of Nottingham, Sutton Bonington, LE12 5RD, UK; 3 Department of Molecular Mechanisms of Mycobacterial Infections, Institut de Pharmacologie et de Biologie Structurale, Centre National de la Recherche Scientifique Unité Mixte de Recherche 5089, Toulouse, France; 4 Present address : School of Crystallography, Birkbeck College, Malet Street, London WC1E 7HX, UK

## Abstract

**Background:**

The cell wall of *Mycobacterium tuberculosis *contains a wide range of phosphatidyl inositol-based glycolipids that play critical structural roles and, in part, govern pathogen-host interactions. Synthesis of phosphatidyl inositol is dependent on free myo-inositol, generated through dephosphorylation of myo-inositol-1-phosphate by inositol monophosphatase (IMPase). Human IMPase, the putative target of lithium therapy, has been studied extensively, but the function of four IMPase-like genes in *M. tuberculosis *is unclear.

**Results:**

We determined the crystal structure, to 2.6 Å resolution, of the IMPase *M. tuberculosis *SuhB in the apo form, and analysed self-assembly by analytical ultracentrifugation. Contrary to the paradigm of constitutive dimerization of IMPases, SuhB is predominantly monomeric in the absence of the physiological activator Mg^2+^, in spite of a conserved fold and apparent dimerization in the crystal. However, Mg^2+ ^concentrations that result in enzymatic activation of SuhB decisively promote dimerization, with the inhibitor Li^+ ^amplifying the effect of Mg^2+^, but failing to induce dimerization on its own.

**Conclusion:**

The correlation of Mg^2+^-driven enzymatic activity with dimerization suggests that catalytic activity is linked to the dimer form. Current models of lithium inhibition of IMPases posit that Li^+ ^competes for one of three catalytic Mg^2+ ^sites in the active site, stabilized by a mobile loop at the dimer interface. Our data suggest that Mg^2+^/Li^+^-induced ordering of this loop may promote dimerization by expanding the dimer interface of SuhB. The dynamic nature of the monomer-dimer equilibrium may also explain the extended concentration range over which Mg^2+ ^maintains SuhB activity.

## Background

The enzyme inositol monophosphatase (*myo*-inositol-1-phosphate phosphohydrolase, EC 3.1.3.25, IMPase) has attracted considerable scrutiny since the early 1980s. Then, it was discovered that low millimolar concentrations of Li^+ ^inhibit IMPase-catalysed dephosphorylation of *myo*-inositol-1-phosphate [1], which could plausibly explain the marked decrease of *myo*-inositol in brain tissue following administration of lithium, a veteran therapeutic in manic depression treatment [2]. Inhibition of *myo*-inositol synthesis affects the phosphatidyl inositol second messenger pathway, which is linked to manic depression, and it is now widely accepted, though unproven, that IMPases constitute a major target of lithium therapy [3].

The mycobacterial cell wall contains several lipid constituents based on the structure of phosphatidylinositol (PI), such as free PI, phosphatidylinositol-mannosides, lipomannan, and lipoarabinomannan [4]. In addition to their critical structural role, these lipids are significant as immunomodulatory factors in interactions of the tubercle bacillus with the host [5-7]. PI, an essential structural component, is synthesised by phosphatidylinositol synthase, *M. tuberculosis *PgsA, from CDP-diacylglycerol and *myo*-inositol [8]. In mycobacteria, the supply of *myo*-inositol to PI synthesis is thought to be maintained by *de novo *synthesis, which entails conversion of glucose-6-phosphate to inositol-1-phosphate, catalysed by inositol-1-phosphate synthase, and subsequent dephosphorylation of inositol-1-phosphate catalysed by an IMPase [9]. Thus, IMPase activity is a critical part of PI biosynthesis in mycobacteria. Indeed, growth of *Mycobacterium smegmatis *is accompanied by IMPase activity that dies off as growth reaches the stationary phase, while growth is retarded in the presence of lithium [10].

**Figure 1:**
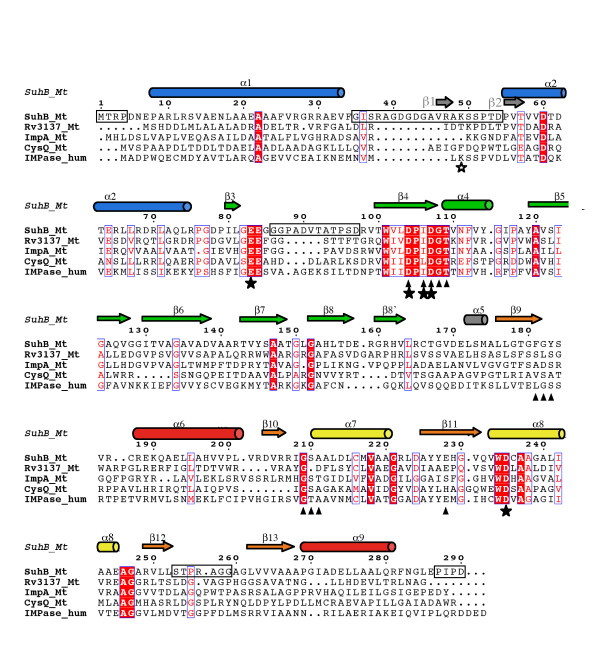
**Sequence comparison of *M. tuberculosis *IMPase-like proteins with human IMPase**. Sequences were aligned based on the structural superimposition of SuhB and human IMPase using STRAP [53], and formatted using ESPript [54]. Secondary structure elements of SuhB are above the sequence, cylinders representing helices and arrows β-strands, coloured according to Fig. 2A. Full asterisks denote residues coordinating the catalytic Mg^2+ ^sites, triangles indicate residues contacting inositol-1-phosphate in the simulated SuhB-substrate complex. Horizontal boxes indicate disordered parts of the sequence. The grey arrows indicate the positions of strands β1, β2 in the α1-α2 loop ('mobile loop') of human IMPase, with the open asterisk marking the critical lysine residue.

The *M. tuberculosis *genome encodes four IMPase-like genes (Figure 1) [11,12], but little is known about the function of the corresponding proteins in terms of *M. tuberculosis *biology. The enzyme most closely related to human IMPase (25% identity), *M. tuberculosis *SuhB (Rv2701c), has been annotated as a 'putative extragenic suppressor protein' according to its homology to *E. coli *SuhB. The *E. coli *enzyme has been implicated in posttranscriptional control of gene expression [13], but no such function has to date been described for *M. tuberculosis *SuhB. According to the transposon mutagenesis study by Sassetti *et al*., Rv3137 is essential, SuhB is dispensable [14], and information on essentiality is as yet unavailable for CysQ and ImpA. *In vitro *analyses showed that *M. tuberculosis *CysQ hydrolyses inositol-1-phosphate, adenosine-monophosphate and fructose-1,6-bisphosphate [15]. In contrast, SuhB appears to be a *bona fide *IMPase with little or no activity towards fructose-1,6-bisphosphate. Still, SuhB also de-phosphorylates a series of polyol phosphates including glucitol-6-phosphate, glycerol-2-phosphate, and 2'-AMP, albeit with significantly reduced efficacy [12]. Like the human orthologue, SuhB is activated by Mg^2+^, and inhibited by lithium. Nevertheless, SuhB requires higher concentrations of Mg^2+ ^for full activation (at ~6 mM), and activity persists over a much wider concentration range (~100 mM) of the activating ion before it becomes inhibitory [12]. In order to better understand potential functional differences between the mycobacterial and eukaryotic IMPases, we have determined the crystal structure of *M. tuberculosis *SuhB and characterised its self-assembly state in solution.

## Results

### Structure determination of SuhB

**Figure 2:**
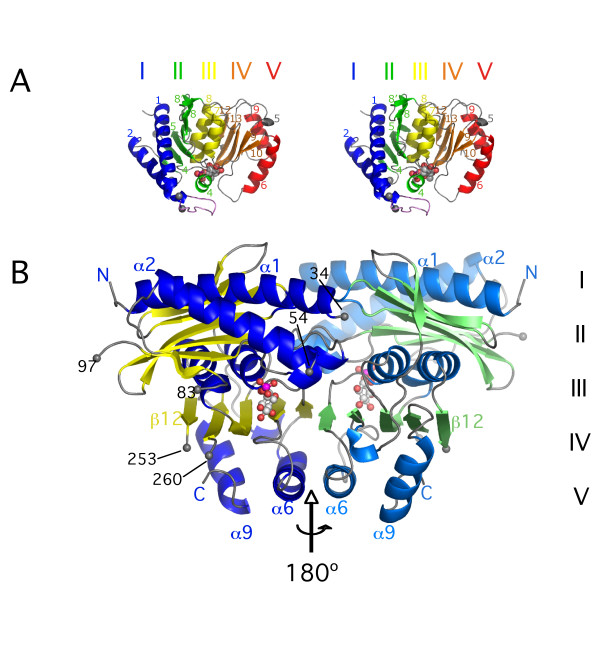
**Fold and crystal dimer of *M. tuberculosis *SuhB**. (**A**) Stereo ribbon diagram of the SuhB monomer, with secondary structure elements coloured according to the penta-layered αβαβα-sandwich arrangement of IMPases (cf. Figure 1). (**B**) Dimer of SuhB formed by subunits A (blue, yellow) and C (lightblue, green). Selected secondary structure elements are labelled for ease of comparison with panel A. Grey spheres indicate boundaries of disordered loops, with corresponding residue numbers in black type. A (modelled) molecule of inositol-1-phosphate in ball-and-stick representation indicates the location of the active site(s) in both panels.

The structure of M. tuberculosis SuhB was determined by molecular replacement to a resolution of 2.6 Å (Table 1, Figure 2). The asymmetric unit of the crystal lattice contains three independent copies of the SuhB monomer forming two crystallographically distinct dimers: chains A and C associate as one dimer, whereas chain B forms an analogous dimer with one of its symmetry mates. The quality of the refined model is limited by several factors: first, a high Wilson B-factor (77.1 Å^2^) of the measured structure factor amplitudes is mirrored by a high average atomic displacement factor (78 Å^2^) (Table 1); second, three disordered loop regions (see below) and mild disorder at either terminus leave 42 of 290 residues of the primary sequence unaccounted for (Figure 1); third, despite a nominal resolution of 2.6 Å and reasonably strong data in the high-resolution shell (Table 1), structural details are less well defined by the (calculated) density than expected at this resolution. In order to counteract an unfavourable observables-to-parameter ratio, non-crystallographic symmetry (NCS) restraints were employed, leading to root mean square (RMS) deviations between NCS-related molecules of 0.05 Å for backbone and ≤ 0.42 Å for side chain atoms. Based on visual impression and real space R-factor, the electron density is best defined for subunits A and C, and worst for subunit B.

**Table 1:** Crystallographic statistics

Beamline	ID14-2 (ESRF, Grenoble)
Wavelength (Å)	0.933
Space group	*C*222_1_
Unit cell parameters	
a, b, c (Å)	101.5, 185.4, 106.9
Resolution range (Å)	20–2.60 (2.69–2.60)^a^
Unique reflections	30931 (3028)
Completeness (%)	98.7 (97.4)
< I/σ(I) >	24.5 (2.5)
Multiplicity	4.9 (3.8)
Rsym^b ^(%)	6.8 (25.8)
Rcryst/Rfree^c ^(%)	22.3/24.5
No. of non-hydrogen atoms	5466
Protein	5342
Solvent	124
Average B-factors (Å^2^)	78
Main chain – subunit A/B/C	68/89/72
Side-chain – subunit A/B/C	71/91/76
Solvent	81
Wilson B-factor	77.1
RMSD bonds (Å)	0.011
RMSD angles (°)	1.26
Ramachandran statistics^d^	
Core	91.4%
Allowed	7.9%
Generous	0.7%
Disallowed	None

^a ^Numbers in parenthesis refer to the outer resolution bin.

^b ^Rsym = Σ_h_Σ_i _| I(h, i) - < I(h) >|/Σ_h_Σ_i _I(h, i), where I(h, i) is the intensity of the ith measurement of reflection h and < I(h) > is the mean value of I(h, i) for all i measurements.

^c ^Rcryst = Σ_hkl_||Fo| - |Fc||/Σ |Fo|, where Fo is the observed structure-factor amplitude and Fc the calculated structure-factor amplitude. Rfree is calculated based on 5% of reflections not used in the refinement.

^d ^The Ramachandran plot was calculated using *PROCHECK *[43]

Apart from minor discrepancies, the SuhB monomer is identical in fold to eukaryotic IMPases [16,17], with RMS deviations between Cα positions of superimposed backbone structures ranging from 1.09 Å to 1.15 Å (Table 2). In order to more readily compare SuhB with related structures we denote secondary structure according to the recent report of the 1.4 Å-resolution structure of bovine IMPase (Figures 1 and 2A) [17]. The IMPase fold is characterised by alternating layers of α-helices and β-sheets in an α-β-α-β-α sandwich arrangement (Figure 2A). Comparative studies established that IMPases share a structural scaffold with inositol polyphosphate-1-phosphatases (IPPases), fructose-1,6-biphosphatases (FBPases), 3'-phosphoadenosine-5'-phosphatases (PAPases) and 3'-phosphoadenosine-5'-phosphatase/inositol-1,4-bisphophatases (PIPases) (Table 2), although on the amino acid sequence level the evolutionary relationship between these groups of enzymes is not obvious [18-20]. However, members of this super-family differ notably with respect to their assembly state: eukaryotic IPPases, PAPases and PIPases are monomeric [19,21,22], eukaryotic IMPases form homodimers as do a subset of dual activity IMPase/FBPases [16,17,23-25], whereas FBPases and the dual activity IMPase/FBPase of *Thermotoga maritima *form tetrameric assemblies [26,27].

**Table 2:** Comparison of *M. tuberculosis *SuhB to selected members of the Mg^2+^-dependent/Li^+^-inhibited phosphomonoesterase family of proteins

	IMPase^c^			FBPase		IMPase/FBPase		PAPase	IPPase	PiPase
PDB ID^a^	1IMA	2CZH	2BJI	1FBP	1DCU	1LBV	1DK4	1QGX	1INP	1JP4

RMSD (Å)	1.09 (197)^b^	1.12 (199)	1.15 (193)	1.61 (159)	1.88 (138)	1.23 (177)	1.13 (158)	1.28 (190)	1.41 (166)	1.40 (182)

^a^References for the pdb entries cited in this table are provided in the supplementary information [see Additional file 3].

^b^Number of aligned Cα positions.

^c^IMPase (inositol-1-monophosphatase), FBP (fructose-1,6-bisphosphatase), PAPase (3'-phosphoadenosine-5'-phosphatase), IPPase (inositol-polyphosphate-1-phosphatase), PIPase (inositol-polyphosphate-1-phosphatase and 3'-phosphoadenosine-5'-phosphate phosphatase)

Eukaryotic adenosine-monophosphate (AMP)-regulated FBPases have been described in terms of two sub-domains. In this description, layers I and II of the α-β-α-β-α sandwich are equivalent to the AMP-binding sub-domain of porcine FBPase, and layers III to V correspond to the fructose-1,6-bisphosphate binding sub-domain (Figure 2A) [26]. The primary sequence runs sequentially through layers I and II of the 'AMP-binding' sub-domain, but meanders in the fructose-1,6-bisphosphate-binding sub-domain between layers III-V, with helices and strands alternating. This pattern is interrupted only for strands β12 and β13, which are not separated by a helix (Figures 1, 2A). The dimer observed in the crystal structure of SuhB is primarily mediated by helices α6 in layer V and the β-sheet in layer IV, which extends across the interface (Figure 2B). In contrast, strands in the sheet of layer II are oriented perpendicular to the plane of the dimer interface.

Due to the absence of divalent metal ions and of substrate in the active site, the loop connecting helices α1 and α2 is disordered (residues 35–53 missing), as is much of the loop connecting strands β3 and β4 (residues 85–96 missing). Both features are consistent with the structure of the *apo *form of human IMPase (pdb entry 1IMF, [28]). Soaks of SuhB crystals in Mg^2+^- or Mn^2+^-containing cryoprotection buffers failed to induce ordering, or give rise to density peaks associated with metals sites known from the eukaryotic orthologues, which we attribute to the acidic pH (4.1–4.5) of the crystallisation buffer. Further disorder was seen in the hairpin loop connecting strands β12 and β13 (residues 254–259) in layer IV, while density for the β9-α6 loop was disjointed, yet indicating that the conformation of the loop between residues 183 and 186 differed markedly between subunits. As a consequence, side chains for residues Tyr183, Val185, Arg186 and Cys187 in this loop could not be built.

### Active Site

The active site of SuhB is situated in a cavity carved out at the N-terminal end of helix α7, sandwiched between layers II and IV (Figure 2A). Due to the disordered α1-α2 loop – referred to as the 'mobile loop' [27] – the active site in SuhB appears wide open to solvent. Yet, it is anticipated that the α1-α2 loop will become ordered upon the enzyme binding metal ions and substrate. In this state the active site is anticipated to be effectively shielded from solvent, as is the case in the ligand-bound structure of human IMPase [29]. In SuhB, the α1-α2 loop has a 10-residue insertion relative to human IMPase (Figure 1). Hence, the exact conformation of this loop in the ordered state may well deviate from that of the human enzyme. Interestingly, alternative secondary structure conformations of this loop were observed within the tetramer of the structure of *Thermotoga maritima *IMPase/FBPase TM1415. While the mobile loop included a short helix in two subunits of the tetramer, it assumed a β-hairpin conformation in the other two subunits [27]. The mobile loop includes a lysine residue at position 36 (corresponding to residue 49 in SuhB, marked by I in Figure 1) that stabilizes the third of three catalytic metal sites through hydrogen bonds to two ordered water molecules. In SuhB, this lysine is conserved and parallels in the characteristics of Li^+ ^inhibition between SuhB and human IMPase (see Discussion) suggest an equivalent pattern of interactions in the mycobacterial enzyme.

**Figure 3:**
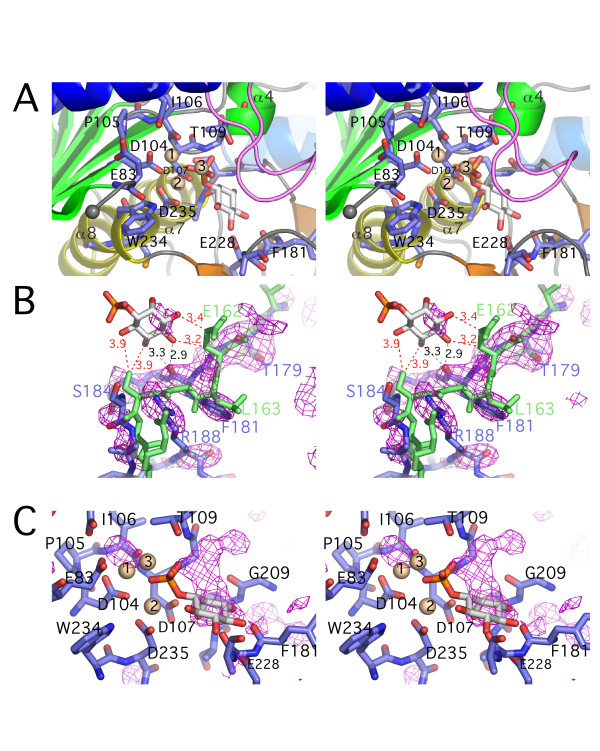
**Stereo views of the active site**. (**A**) Active site of subunit A of SuhB, with metal sites (beige) and inositol-1-phosphate (coloured by atom type, carbons in light grey) modelled based on superimposition with the structures of bovine (PDB:2BJI, [17]) and human IMPase (PDB:1IMA, [29]). Residues in contact with metal sites and substrate in the modelled complex are shown as sticks and numbered according to the SuhB sequence. Secondary structure elements are coloured as in Figure 2A. The loop in violet indicates the approximate position of the α1-α2 loop, based on the superposition with PDB:1IMA [29]. (**B**) Bias-free difference density map of the β9-α6 loop in SuhB, calculated after simulated annealing of the model with residues 178–188 deleted and contoured at 2σ. The density is superimposed with *apo *SuhB (blue sticks) and human IMPase in complex with inositol-1-phosphate (light green, PDB:1IMA, [29]). Putative (grey) and experimental (red) contact distances with substrate are indicated. (**C**) Active site of subunit C of SuhB with σ_A_-weighted Fo-Fc map contoured at 3σ, showing unexplained density around the putative substrate position and metal site 1.

Structural elements of the active site required for metal and substrate binding include the IMPase signature motif ^104^DPXDGT^109 ^(superscripts denote residue numbers of the SuhB sequence) between strand β4 and helix α4, the ^82^GEEG^85 ^motif at the 'tip' of the β3-β4 loop, the ^209^G [ST]AA^212 ^motif in the β10-α7 loop, in addition to Glu228 in β11 and the ^234^WDXA^237 ^motif in helix α8. All elements contributing to metal site coordination are contained within one subunit of the dimeric assembly. Compelling evidence for three sites occupied by Mg^2+ ^was provided only recently by the 1.4 Å-structure of bovine IMPase [17]. This study, in conjunction with preceding structural studies on lithium-sensitive PAPase and lithium-insensitive IMPase/FBPases [21,24,25,27], provided strong support for a 3-metal catalytic reaction mechanism. When mapped onto the structure of SuhB, these three magnesium ion sites are coordinated by Glu83 (equivalent to residue 70 in human/bovine IMPase: h_70), Asp104 (h_90), Asp107 (h_93), Asp235 (h_220), in addition to the carbonyl of Ile106 (h_92). In a previous study we had shown that mutations to these sites in SuhB reduced activity dramatically [12]. Likewise, when activity was tested in the presence of various divalent cations, Mg^2+ ^was the most potent activator of SuhB. These data and the high level of sequence conservation among active site residues justify the attempt to construct a model of inositol-1-phosphate in the active site of SuhB (Figure 3A) and to analyse potential differences in substrate-enzyme contacts.

In our model of the SuhB-substrate complex, the phosphate moiety is positioned at the N-terminal end of helix α4, the helix dipole countering the charge of the phosphate (Figure 3A). Most contacts between enzyme and phosphate are through the three Mg^2+ ^ions, with contact distances in the order of 2.15–2.3 Å, in agreement with the experimental structures of the eukaryotic IMPases [17,28,29]. In addition, the phosphate forms H-bond interactions with the amide nitrogen of Gly108 (h_94) and Thr109 (h_95) at the N-terminus of helix α4. The inositol moiety packs primarily against residues in the β9-α6 and β10-α7 loops with specificity-determining contacts provided by Glu228 (h_213) in strand β11, and Asp107 (h_93) in the β4-α4 loop (Figure 3A). All conserved side chains forming the metal binding sites or contacting inositol-1-phosphate are seen in essentially the same conformation as in the human/bovine enzyme, with the exception of Glu83: as a consequence of disorder in a large part of the β3-β4 loop, Glu83 (h_70) in subunit A points outward rather into the active site (Figure 3A).

Significant discrepancies between the *M. tuberculosis *and the human enzyme are seen in the β9-α6 loop, which in human IMPase provides a contact surface, but no specificity-determining interactions with the inositol ring [29] (Figure 3B). The β9-α6 loop in SuhB is two residues shorter than in the human enzyme with weak sequence conservation (Figure 1). Although poorly ordered between residues 183–186, residues in proximity to the putative position of the inositol ring – Gly180, Phe181 and Gly182 – are well defined by density (Figure 3B). Interestingly, the carbonyl oxygen of Phe181 falls within H-bonding distance range of the C3 (2.9 Å) and C4 (3.3 Å) hydroxyls of the inositol ring, suggesting that the β9-α6 of SuhB may form specificity-determining contacts to the substrate that are not present in the human enzyme.

A surprising observation was a patch of unexplained density in the active site that overlaps significantly with the modelled position of inositol-1-phosphate, while a second density peak coincides with the metal site 1 (Figure 3C) (in the numbering of ref. [17]). The latter is situated between the carbonyl oxygen of Ile106 and the carboxylate of Asp104 and corresponds to the high-affinity metal site [17]. The 'metal site 1' peak was present in all three active sites, whereas the density for the inositol-1-phosphate site was best defined in subunit C (Figure 3C). We consider it likely that the observed density represents a weakly-bound molecule of octyl β-D-glucopyranoside, a reagent used as an additive during crystallisation. Whether or not the density peak a metal site 1 represents a divalent cation could not be verified.

### Dimer interface and assembly state

**Figure 4:**
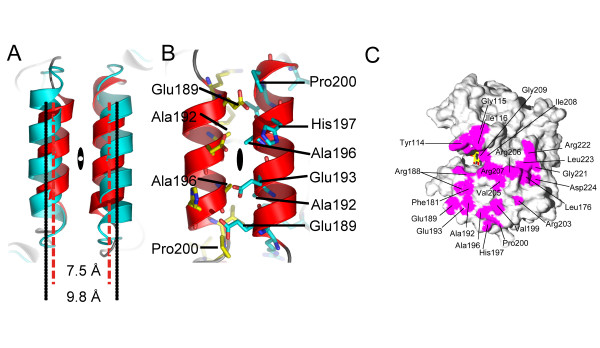
**Analysis of dimer interface**. (**A**) Helices α6 after superimposition of the dimers of human IMPase (cyan) and SuhB (red) by secondary structure matching. The spacing of the helix axes is indicated in units of Å and the black oval indicates the position of the non-crystallographic 2-fold axis mapping the subunits onto each other. (**B**) The helix α6 interface in *SuhB *with side chains contributing to the contact surface indicated as sticks in cyan (subunit A) and yellow (subunit C). (**C**) Contact surface (magenta) of subunit A calculated and visualised using Swiss PDB Viewer [50]. The active site indicated by a molecule of inositol-1-phosphate (yellow sticks).

The present crystal structure comprises two apparent dimers of SuhB. One dimer is formed between the NCS-related subunits A and C, while the second dimer results from crystallographic symmetry, pairing two symmetry-related copies of subunit B. As a result of NCS restraints applied during the refinement no substantial difference between the two independent dimers is observed at the interface. The SuhB dimers also superimpose closely with the dimers of the human and bovine IMPase enzymes, whereby the dimer interface is formed by analogous secondary structure elements. These include helix α6, the strand β10, the β9-α 6 loop, helix α4, and the β10-α7 and α7-β11 loops. In the superposition, which is based on secondary structure matching, helices α6 of the two protomers are positioned opposite to each other (Figures 2B, 4A), contributing approximately 30% of the buried surface area (per monomer). It seems noteworthy that in SuhB the axes of these two helices are spaced 7.5 Å apart whereas in the eukaryotic enzymes that spacing is approximately 10 Å (Figure 4A). The discrepancy can be attributed to alanine residues 192 and 196 being positioned at the centre of this interface (Figure 4B), while the side chains of a leucine at position 176 occupy the corresponding space in human IMPase.

The interface of the SuhB dimer buries a total of 2696 Å^2 ^of solvent accessible surface, calculated using the *PISA *interface server [30,31], or 1348 Å^2 ^per monomer. In terms of buried surface area hydrophobic interactions clearly dominate over H-bonds (12 contacts). Although the residues located at the interface include a number of polar and charged side chains (Figure 4C) only one ionic interaction is seen. In human IMPase distinctly more solvent accessible surface is buried in the interface (1675 Å^2 ^per monomer). Contacts across the interface include 11 ionic interactions, while the number of H-bonds (13) is about the same as in SuhB. One factor explaining the difference in size of the interface, at least in part, is the disordered 'mobile loop' in SuhB. In the *apo *structure of human IMPase (1IMF – [29]), the α1-α2 loop is also disordered, reducing the total of buried solvent accessible surface per monomer from 1675 Å^2 ^to 1571 Å^2^. Thus disorder of this loop accounts partially for the observed discrepancy.

**Figure 5:**
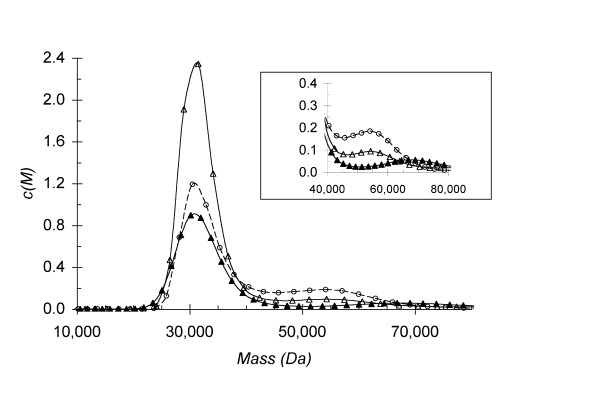
**Analytical ultracentrifugation in sedimentation velocity mode demonstrates that SuhB is monomeric in the absence of Mg^2+^**. Main panel: Traces of the molecular weight distribution of *M. tuberculosis *SuhB in a sedimentation velocity experiment. Conditions analysed were 0.5 mg.ml^-1 ^SuhB (solid triangle), 1.0 mg.ml^-1 ^SuhB (open triangle),1.0 mg.ml^-1 ^SuhB + 1 mM Mg^2+ ^(open circle). The main peak is centred at 30,508 Da, the secondary peak at 54,160 Da. Inset: enlarged view of the region between 40,000 and 80,000 Da. Samples were centrifuged at 40,000 rpm, 4°C, for a minimum of 12 hours, in 20 mM Tris-HCl pH 7.9, 50 mM NaCl and MgCl_2 _as indicated.

The analysis of the dimer interface using the *PISA *server suggested that dimerisation of SuhB might not be constitutive. Based on molecular contacts at the interface, *PISA *calculates a 'complexation significance score' (CSS) that on a scale from 0 to 1 indicates the probability that a packing interface generated by crystal symmetry might represent a 'real' interface [31]. The CSS for the dimer interface of SuhB is 0.1, compared to a score of 1.0 for the dimer interface of human IMPase (with or without the α1-α2 loop in the ordered state), suggesting that dimerisation was merely due to crystal packing. We analysed self-association of SuhB by analytical ultracentrifugation (AUC) in sedimentation velocity mode. To our surprise, but consistent with the prediction by *PISA*, we found that SuhB is predominantly monomeric, with the peak at molecular weight 30,508 Da closely matching the calculated mass of the SuhB monomer (Figure 5). However, with increasing protein concentration a second peak at molecular weight of 54,160 Da appears, and this peak becomes more pronounced, at the expense of the main peak, when 1 mM Mg^2+ ^is added to the buffer (Figure 5). We verified by SDS gel electrophoresis that the preparation of SuhB used in this experiment did not contain a contaminant that could explain the peak at 54,160 Da (data not shown).

**Figure 6:**
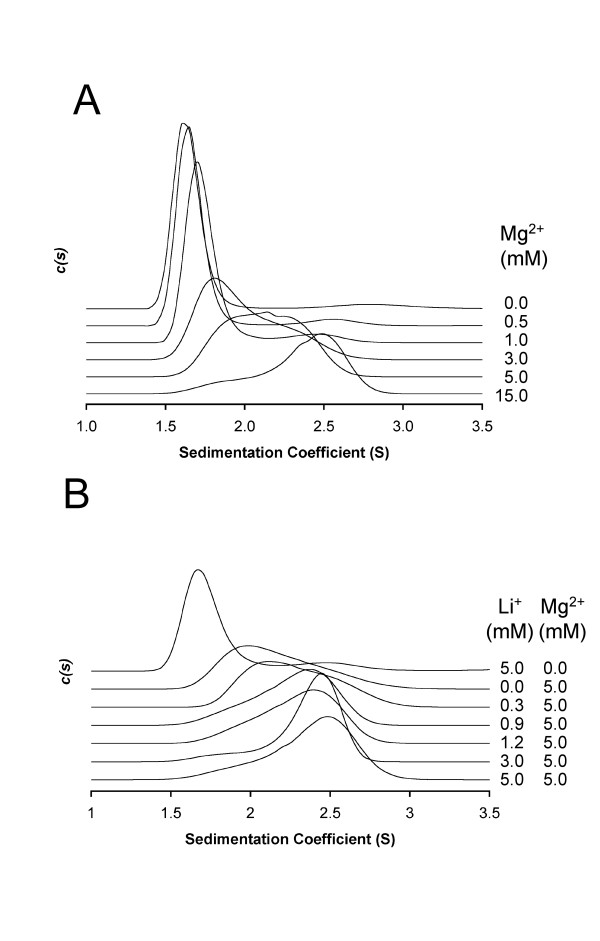
**Dimerization of SuhB is induced by Mg^2+^, or Mg^2+ ^and Li^+^, but not by Li^+ ^alone**. Traces of the sedimentation coefficient distribution recorded in sedimentation velocity experiments of *M. tuberculosis *SuhB. SuhB was at 1.0 mg.ml^-1 ^in 20 mM Tris-HCl pH 7.9, 50 mM NaCl, plus MgCl_2 _or LiCl as indicated. Samples were centrifuged at 40,000 rpm at 4°C for at least 12 hours. The peak at 1.6 *S *corresponds to a molecular weight of 30,147 Da, the peak at 2.5 *S *corresponds to 50,116 Da.

These data suggested that SuhB, at protein concentrations in the order of 1 mg.ml^-1^, exists in a monomer-dimer equilibrium, but that Mg^2+^, which is required for activity, might shift self assembly to the dimer state. The underestimate in molecular weight of the dimer peak is due to the fitting of a single frictional coefficient to two species that have differing frictional ratios. In addition, the appearance of two separate peaks in the molecular weight profile with increasing concentration indicates that the off-rate for dimerisation is slow compared with the time of sedimentation. Values for the off-rate for this regime are therefore estimated to be 10^-5 ^< k_off _< 10^-3 ^[32]. Analysing the sedimentation velocity of SuhB in the presence of increasing concentrations of Mg^2+^, confirmed that in the absence of Mg^2+ ^the monomer form is strongly preferred (Figure 6A). Yet, as Mg^2+ ^increases in concentration (at a protein concentration of 1.0 mg.ml^-1^) two features are observed: first, the dimer peak increases in height, at the expense of the monomer peak; second, the monomer and dimer peaks shift towards each other until, at 5 mM Mg^2+^, a broad skewed distribution of the sedimentation coefficient is observed (Figure 6A), suggesting a gradual transition from slow to fast exchange between the two assembly states due to an increase in the off-rate of dimerisation. Increasing the concentration of Mg^2+ ^further, the dimer peak eventually becomes the dominant species in the c(*S*) distribution (Figure 6A).

Next, we examined whether the inhibitor Li^+ ^influenced dimerization. At an enzyme concentration of 1.0 mg.ml^-1 ^and in the presence of 5 mM Mg^2+^, increasing concentrations of Li^+ ^amplify the effect of Mg^2+^-induced dimerization, with the dimer peak becoming dominant over the monomer peak (Figure 6B). However on its own Li^+ ^promotes dimerization only very weakly, if at all (Figure 6B). Furthermore the c(*S*) distribution obtained for 15 mM Mg^2+ ^matches almost perfectly the one obtained in presence of 5 mM Mg^2+ ^and 5 mM Li^+^. Calcium, which binds to IMPase on identical sites as Mg^2+^, but does not activate, strongly promotes dimerization, while EDTA reverses Mg^2+^-induced dimerization [see Additional file 1].

## Discussion

The present crystal structure of *M. tuberculosis *SuhB confirms a highly conserved structural scaffold of IMPases in mycobacteria with respect to overall fold and active site geometry, in spite of a low level of overall sequence identity (25%) relative to human IMPase. The structural similarity correlates with similar biochemical characteristics – activation by magnesium, inhibition by lithium, specificity for inositol-1-phosphate and exclusion of fructose-1,6-bisphosphate as a substrate. It has been noted previously that the Mg^2+^-dependence of IMPase activity of SuhB resembles more closely that of the thermophilic species *Thermotoga maritima*, *Archeoglobus fulgidus *and *Methanococcus jannaschii *[12,25,33,34] in that activation is maintained over a concentration range of Mg^2+ ^in the order of 100 mM, whereas in eukaryotic IMPases Mg^2+ ^becomes inhibitory above 5 mM. Such comparison ignores, however, that unlike these thermophilic enzymes SuhB does not hydrolyze fructose-1,6-bisphosphate, and remains sensitive to Li^+ ^(IC_50 _0.9 mM, [12]). Given that the active site of SuhB, in its 'non-mobile' part, displays the same highly conserved framework of side chains coordinating the three metal sites as the eukaryotic orthologues, we postulate that differences in Mg^2+^-dependence of activity between SuhB and eukaryotic IMPases must be linked on the one hand to structural differences in the mobile α1-α2 loop, and secondly to the apparent link between Mg^2+^-driven dimerization and activation of SuhB. In IMPases and the tetrameric IMPase/FBPases the α1-α2 loop, when ordered, stabilizes the third of the three catalytic metal sites through bridging water molecules [17,25,29]. The α1-α2 loop contributes significantly to the dimer interface. This is apparent when comparing the buried solvent-accessible surface in the dimer interface of human IMPase between the ordered and disordered state of the mobile loop. Thus, we anticipate that ordering of the α1-α2 loop in SuhB, which is 10 residues longer compared to human IMPase, will expand the dimer interface and help stabilize the dimer state. While SuhB is driven to the dimer state at high protein concentrations in the absence of metal ions, as was the case during crystallization, it is evident that Mg^2+ ^strongly promotes dimerization at low concentrations. Lithium amplifies the Mg^2+^-induced effect, yet Li^+ ^alone does not noticeably shift the monomer dimer equilibrium. These observations correlate with the characteristics of metal binding in the active site, which is known to promote ordering of the α1-α2 loop. According to ^7^Li-NMR binding data, lithium occupies a single site per monomer in IMPase [35], and various indirect evidence points to Li^+ ^displacing Mg^2+ ^from either site 2 or site 3 [17,21,36,37]. A lysine residue in the mobile loop (Lys36 in human, open asterisk in Figure 1) that coordinates the third metal site via hydrogen bonds to bridging water molecules has been shown to critically influence Li^+^-sensitivity [37], and this lysine is conserved in SuhB (Lys49). Given that the α1-α2 loop is disordered, the present structure leaves open the precise geometry of this loop in the ordered state and whether Lys49 in SuhB coordinates the third metal site. Yet, lithium sensitivity of SuhB (IC_50 _0.9 mM, [12]), the debilitating effect on activity of the W234A mutation, and the loss of Li+ inhibition through the L81A mutation [12] – a mutation that removes a stabilising hydrophobic contact to the conserved Trp234 in the active site – hint that metal binding induces ordering of the α1-α2 loop in SuhB in a similar fashion as in the eukaryotic orthologues.

Previously, size exclusion chromatography experiments with 1 mM EDTA present in the buffer had indicated that *E. coli *SuhB formed monomers and it had been inferred that the *E. coli *enzyme was active in the monomeric form [13]. The parallels between metal-dependent self-association and enzymatic activation in *M. tuberculosis *SuhB strongly suggest that dimerization is linked to loading of the three metal sites in the active site, and that phosphatase activity occurs in the dimer state, although we are not in a position to determine whether dimerization is required for activity. If dimerization were required for enzymatic activity of SuhB, this would at least in part explain the wide range of Mg^2+ ^concentrations over which activity is maintained, as the transition to the dimer state is not complete up to at least 15 mM Mg^2+^. Several *caveats *go with this rationale. First, the comparison of the activity and AUC data is complicated by the fact that metal binding is cooperative with substrate binding [38] and inositol-1-phosphate was not present in the AUC experiments. Also, while dimerisation appears to correlate with loading of the metal positions in the active site, we cannot rule out the possibility that the dimer interface contains one or more metal binding sites, which could drive dimerisation. However, the present structure, in line with related IMPases, and a series of metal-soaking experiments with SuhB crystals provided no indication for such a site (data not shown).

Crystal structures of eukaryotic IMPases, a refolding study and AUC analysis of human IMPase 2, in the absence of Mg^2+^, consistently indicate constitutive dimerization of the eukaryotic orthologue [16,17,23,39]. It is not clear what mechanistic purpose dimerization serves with respect to the enzymatic properties of IMPases, and why dimerization should be constitutive in eukarya, but not in bacteria. Unlike the tetrameric FBPases, which are regulated by an allosteric mechanism involving changes in the relative orientation of the subunits in the tetramer (see [40] and references therein), no regulatory mechanism for IMPase has been reported that invokes dimerization of the enzyme. Nevertheless, Mg^2+ ^has been shown to moderately enhance thermal stability of *M. tuberculosis *SuhB, which correlates to some extent with phosphohydrolase activity of SuhB peaking at about 80°C [12]. While this latter property is mirrored by *E. coli *SuhB [13] and *M. tuberculosis *CysQ [15] it has not been tested whether at such high temperatures SuhB still discriminates between substrates. Thus, while dimerization may increase thermal stability, the functional role of Mg^2+^-induced dimerization of SuhB in the physiological regime of the tubercle bacillus is not clear.

### SuhB – a template for the other M. tuberculosis IMPases?

The IMPase-like genes in M. tuberculosis display considerable sequence diversity relative to each other. In the multiple sequence alignment (Figure 1), SuhB and Rv3137 align with 30% sequence identity, while ImpA and CysQ score significantly lower (= 23%) relative to any sequence in Figure 1 (Table 3). Searching the PDB for homologues of known structure, using BLAST, showed that SuhB still represents the best template in terms of sequence identity, although the differences to the next nearest homologue are small (Table 3). Based on the search results, one could speculate on the substrate specificities of the two uncharacterised gene products, Rv3137 and ImpA. The gene product with the most divergent sequence, CysQ, has been characterised as a dual IMPase/FBPase [15]. Consistent with this finding, searching the PDB with the CysQ sequence returns two dual activity enzymes, *Rattus norvegicus *3'-phosphoadenosine-5'-phosphate/inositol-1,4-bisphosphate phosphatase (RnPIP) [22], and *T. maritima *IMPase/FBPase TM1415 [18,27] as the closest homologues of known structure. Likewise, the search results mirror the close relationship of SuhB to the eukaryotic IMPases, which strongly prefer inositol-1-phosphate as substrate. Interestingly, the dual activity IMPase/FBPase TM1415 appears as the top hit for both ImpA and Rv3137 (Table 3).

**Table 3:** BLAST search for M. tuberculosis IMPase homologues of known structure

	SuhB	Rv3137	ImpA	CysQ	1IMA^a^	2BJI	1JP4	2P3N	1G0H
SuhB	100	30	23	22	25	25	14	23	19
Rv3137		100	23	18	23	22	22	29	17
ImpA			100	13	15	16	12	22	20
CysQ				100	14	12	16	7	3
1IMA (IMP human)	**2.0E-20**				100	88	16	28	19
	**(194)**								
2BJI (IMP bovine)	**3.0E-21**	**4.7E-07**				100	19	28	20
	**(194)**	**(165)**							
1JP4 (PIPase_Rno)				**2.7E-05**			100	18	15
				**(169)**					
2P3N(TM1415_Tma)		**9.5E-16**	**4.0E-12**	**8.7E-04**				100	23
		**(221)**	**(247)**	**(192)**					
1G0H (MJ0109_Mja)			**6.3E-10**						100
			**(161)**						

Fields above the table diagonal give the %-identity in a *STRAP*-generated multiple sequence alignment. Fields in bold type give the E-values and (number of paired amino acid residues) of the two top hits in the *BLAST *search of the PDB [48].

^a ^References for PDB entries:

1IMA – human IMPase complexed with *myo-*inositol-1-phosphate [29]

2BJI – bovine IMPase [17]

1JP4 – *Rattus norvegicus *3'-phosphoadenosine 5'-phosphate and inositol-1,4-bisphosphate phosphatase [22]

2P3N – *Thermotoga maritima *TM1415 IMPase/FBPase [27]

1G0H – *Methanococcus jannaschii *MJ0109 IMPase/FBPase [24].

Analysing the multiple sequence alignment that underlies Table 3 [see Additional file 2] more closely, it is interesting to note that enzymes that are specific for inositol-1-phosphate and exclude fructose-1,6-bisphosphate carry a glutamic acid or glutamine at position 228 (SuhB sequence; residue 213 in human IMPase), whereas Asp, His, Ser or Thr are found in enzymes that hydrolyze both substrates or are specific for fructose-1,6-bisphosphate. In the inositol-1-phosphate-bound structure of human IMPase [29], the corresponding residue, Glu213, forms hydrogen bonds to the C3 and C4 hydroxyls of the inositol ring. In addition to the H-bond between Asp93 (Asp107 in SuhB) and the C6-hydroxyl, these H-bonds are the only side chain-mediated, specificity-determining contacts between the inositol moiety and the enzyme. In the structural superimposition it is evident that an Asp (and likewise Ser, Thr or His) at this position is unlikely to confer selectivity as the distance between the terminal group of the side chain and the sugar hydroxyls becomes too large. Discrimination against fructose-1,6-bisphosphate is rooted primarily in steric clashes of the 6-phosphate the β9-α6 loop. As a result of deletions relative to human IMPase, a much shorter β9-α6 loop in FBPases and dual activity IMPase/FBPases provide the space to accommodate the second phosphate group. Thus, deletions in this region in addition to substitutions of Glu/Gln at position 228 could be indicative of dual specificity. Analysing the sequences of ImpA and Rv3137 in light of these considerations suggests that at least Rv3137 is restricted to inositol-1-phosphate, whereas the case is less clear cut for ImpA. The latter carries a Ser at the position corresponding to Glu228, but displays no significant deletion relative to SuhB between residues 150 and 190, whereas CysQ by the standards of both criteria falls into the category of the dual specificity enzymes, consistent with published biochemical data [15].

## Conclusion

We have determined the structure of M. tuberculosis SuhB, providing a structural template for the four IMPase-like enzymes in this organism. While resembling eukaryotic IMPases in terms of structural scaffold, specificity for inositol-1-phosphate, requirement for Mg^2+ ^and inhibition by Li^+^, SuhB clearly diverges from the paradigm of constitutive dimerization of *bona fide *IMPases. The present data support a model of Mg^2+^-dependent dimerization, where loading of the three catalytic metal sites in the active site induces ordering of the mobile loop, promoting dimerization likely by expanding the dimer interface. The active site of SuhB presents a highly conserved scaffold of side chains forming the metal- and substrate-binding site, yet additional specificity-determining contacts are expected with the weakly conserved β9-α6 loop. Sequence and structural comparisons lead us to predict that the essential gene product Rv3137 represents a bona fide IMPase, while such may or may not be the case for ImpA.

## Methods

### Materials

Centricon YM-10 filtration units were obtained from Millipore. *E. coli *C41(DE3) [41], acquired from Avidis (France), was used for protein production in this study. Sparse matrix crystallisation screens were obtained from Molecular Dimensions Ltd. All other chemicals were reagent grade or better and obtained from Sigma-Aldrich.

### Expression, purification and crystallisation

Recombinant proteins were generated and purified as described previously [12]. Purified His_6_-tagged SuhB, was dialyzed extensively against 20 mM sodium phosphate pH 7.5, 250 mM NaCl and purified further by size exclusion chromatography over a Sephacryl S200-HR matrix. Pure fractions were pooled and concentrated using Centricon YM-10 filter units. Crystals of SuhB were obtained by hanging drop vapour diffusion, mixing 1 μl of protein solution (40–50 mg.ml^-1^) with 1 μl of reservoir solution, screening against commercial sparse matrix screens (Structure Screen 1 and 2, Molecular Dimensions Ltd.) at 18°C. Initial crystals appeared in 30%(v/v)2-methyl-2, 4-pentanediol(MPD), 0.1 M tri-sodium citrate, pH 5.6, 0.2 M ammonium acetate. Optimization led to crystals approximately 0.3 mm in length at 10–14%(v/v) MPD, 0.1 M citric acid, pH 4.2, and 0.05%(w/v) octyl β-D-glucopyranoside. Crystals grew to their final size over 7–10 days. In preparation for X-ray data collection crystals were soaked in cryoprotectant , consisting of upto 30%(v/v) MPD, 125 mM NaCl, 0.1 M citric acid pH 4.2 and 0.05%(w/v) octyl β-D-glucopyranoside, increasing the MPD concentration in steps of 10%. Crystals were mounted in nylon loops and frozen in a 100 K nitrogen gas stream.

### X-ray data collection and crystallographic analysis

X-ray diffraction data for SuhB were recorded on beamline ID14-EH2 at the European Synchrotron Radiation Facility (ESRF), Grenoble, France (Table 1). Data were reduced using *DENZO/SCALEPACK *[42]. Patterson self-rotation analysis (*POLARRFN *– [43]) revealed a 3-fold non-crystallographic rotation axis parallel to the 2_1_-screw axis (resulting in an apparent 6-fold), consistent with three SuhB monomers per crystallographic asymmetric unit. Structure factor amplitudes were normalized (*ECALC *– [43]), and initial phases were obtained by molecular replacement (*AMORE *– [43]), using monomer coordinates of human IMPase (PDB:1IMA – [29]) as a search model. While the Patterson cross-rotation search did not reveal clear solutions, the correct 3 orientations (peaks 4, 5 and 7) could be identified through comparing rotation angles between rotation function peaks with self-rotation peaks, which was confirmed through the subsequent translation search. The search model was stripped of non-conserved side chains and insertions, and built manually into MR-phased density (*O *– [44]). Search model bias was minimised by building into simulated annealing omit maps [45], followed by rounds of refinement (conjugate-gradient minimization, simulated annealing) in *CNS *ver 1.1 [46] and manual rebuilding, eventually leading to a model that fitted the data with an Rfree of ~30% (5% of reflections). Non-crystallographic symmetry restraints were introduced and the refinement was continued using *REFMAC5 *[47]. The final model contains 3 monomers covering SuhB residues 5–34, 54–85, 98–253, 260–286. Details of the refinement statistics are listed in Table 1. Coordinates and structure factors have been deposited in the Protein Data Bank [48] [PDB:2Q74].

### Molecular graphics

Figures 2, 3 and 4A,4B were prepared using *PyMOL *ver 0.97 [49]. Figure 4C was generated using *Swiss PDB Viewer *[50] and *POV-Ray *Version 3.6 [51].

### Analytical ultracentrifugation

Sedimentation velocity experiments were performed using a Beckman Optima XL-A analytical ultracentrifuge equipped with absorbance optics. Protein samples were dialysed into storage buffer, as indicated in the figure legends (Figures 5 and 6) and loaded into cells with two channel Epon centre pieces and quartz windows. Data were recorded at 40,000 rpm, 4°C. A total of 100 absorbance scans (280 nm) were recorded for each sample, representing the full extent of sedimentation of the sample. Data analysis was performed using the SEDFIT software fitting a single friction coefficient [52].

## Authors' contributions

A.K.B., G.S.B. and K.F. designed the study. A.K.B. wrote the initial draft of the manuscript, and purified the protein. G.M. performed crystallisation experiments, data acquisition and contributed to crystallographic analysis and model building. A.K.B. and H.G. performed the AUC experiment, and with help from D.J.S., analysed the data, and prepared figures. L.G.D. together with J.N. designed and produced the expression plasmids, developed the purification protocol and contributed to data interpretation. K.F. solved the structure, contributed to design of figures, and edited the manuscript. All authors have read and approved the manuscript.

## Supplementary Material

Additional file 1 **Supplementary Figure S1**. This Figure shows traces of the sedimentation coefficient distribution recorded in sedimentation velocity experiments of *M. tuberculosis *SuhB. SuhB was at 1.0 mg.ml^-1 ^in 20 mM Tris-HCl pH 7.9, 50 mM NaCl, plus MgCl_2_, LiCl, CaCl_2 _and EDTA as indicated. Samples were centrifuged at 40,000 rpm at 4°C for at least 12 hours. Click here for file

Additional file 2 **Supplementary Figure S2**. This Figure shows a structure-based sequence alignment of IMPase-like proteins using *STRAP *[53] with formatting in *ESPRIPT *[54]. Sequence abbreviations (with pdb accession code in parentheses) are as follows: SuhB_Mtb – *Mycobacterium tuberculosis *SuhB ; Rv3137_Mtb – gene product Rv3131 of *M. tuberculosis; *ImpA_Mtb – *M. tuberculosis *ImpA; CysQ_Mtb – *M. tuberculosis *CysQ; IMPase_hum – human inositol monophosphatase (1IMA); IMPase2_hum – human inositol monophosphatase 2 (2CZH); IMPase_bov – bovine inositol monophosphatase (2BJI); SuhB_Eco – *Escherichia coli *SuhB; MJ0109_Mja – *Methanococcus jannaschii *IMPase/FBPase MJ0109 (1DK4); AF2372_Afu – *Archeoglobus fulgidus *IMPase/FBPase AF2372 (1LBV); TM1415_Tma – *Thermotoga maritima *IMPase TM1415 (2P3N); PIPase_Rno – *Rattus norvegicus *3'-phosphoadenosine 5'-phosphate and inositol 1,4-bisphosphate phosphatase (1JP4). Secondary structure elements above the sequence refer to the crystal structure of *M. tuberculosis *SuhB. Click here for file

Additional file 3 **References for Table **2. This file contains references for PDB entries cited in Table 2 of the main text. Click here for file
