# Supplementary figures and images for: Dimerization of inositol monophosphatase Mycobacterium tuberculosis SuhB is not constitutive, but induced by binding of the activator Mg2+
Source: BMC Struct Biol. 2007 Aug 28;7:55. doi: 10.1186/1472-6807-7-55 (PMC2080633; doi:10.1186/1472-6807-7-55)

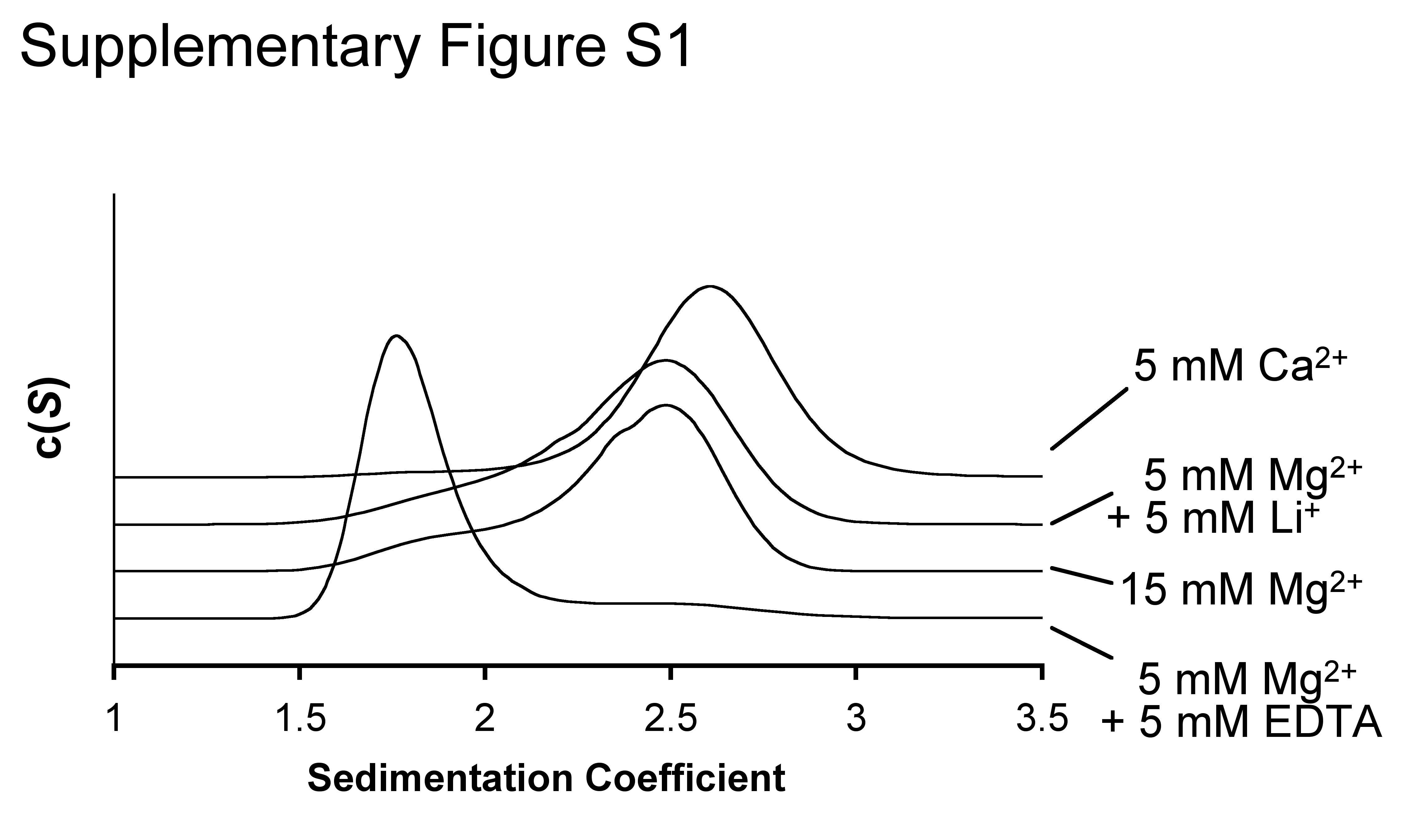

Supplement: Additional file 1 — Supplementary Figure S1. This Figure shows traces of the sedimentation coefficient distribution recorded in sedimentation velocity experiments of M. tuberculosis SuhB. SuhB was at 1.0 mg.ml-1 in 20 mM Tris-HCl pH 7.9, 50 mM NaCl, plus MgCl2, LiCl, CaCl2 and EDTA as indicated. Samples were centrifuged at 40,000 rpm at 4°C for at least 12 hours. [file 1472-6807-7-55-S1.png]

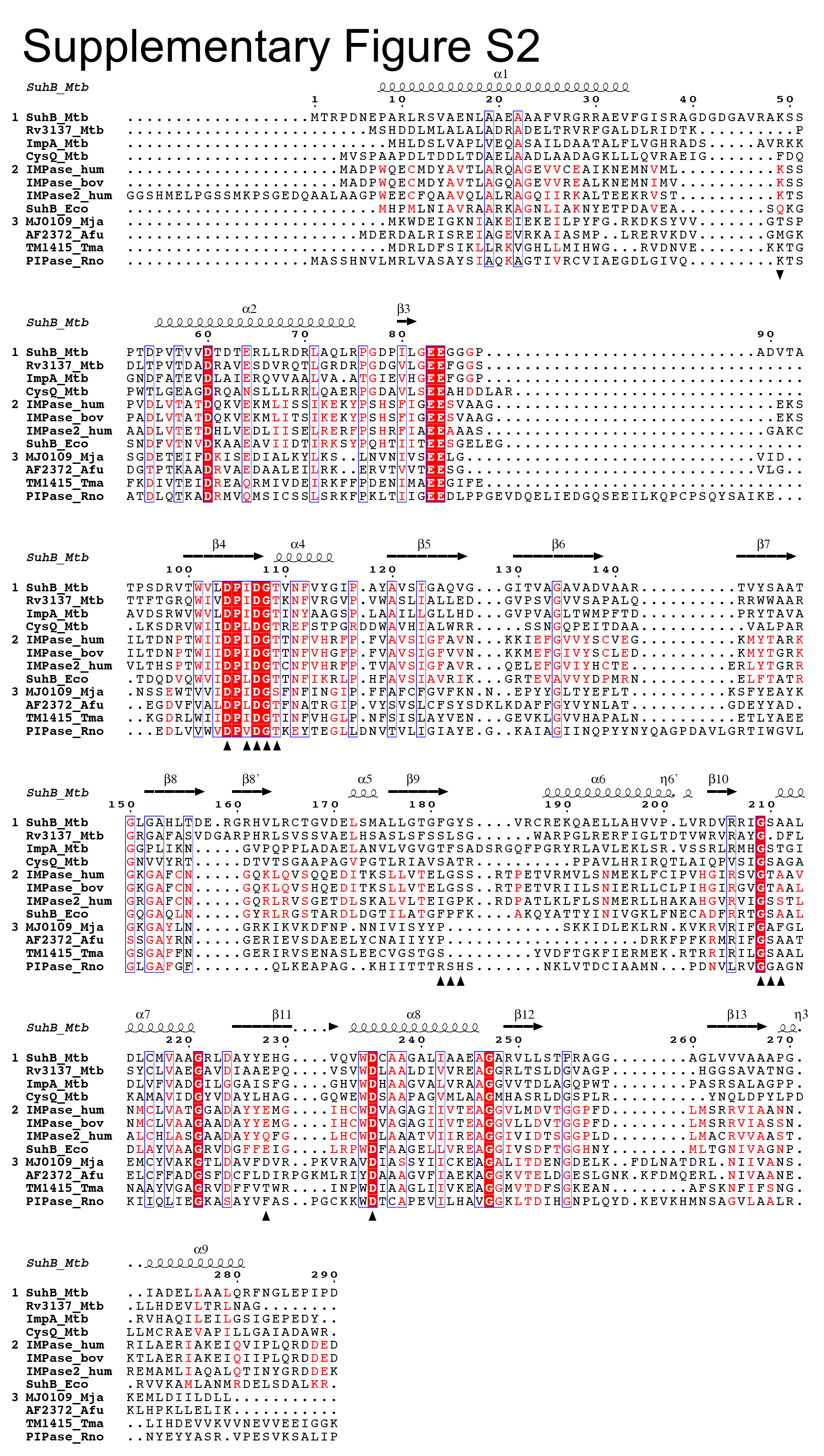

Supplement: Additional file 2 — Supplementary Figure S2. This Figure shows a structure-based sequence alignment of IMPase-like proteins using STRAP [53] with formatting in ESPRIPT [54]. Sequence abbreviations (with pdb accession code in parentheses) are as follows: SuhB_Mtb – Mycobacterium tuberculosis SuhB ; Rv3137_Mtb – gene product Rv3131 of M. tuberculosis; ImpA_Mtb – M. tuberculosis ImpA; CysQ_Mtb – M. tuberculosis CysQ; IMPase_hum – human inositol monophosphatase (1IMA); IMPase2_hum – human inositol monophosphatase 2 (2CZH); IMPase_bov – bovine inositol monophosphatase (2BJI); SuhB_Eco – Escherichia coli SuhB; MJ0109_Mja – Methanococcus jannaschii IMPase/FBPase MJ0109 (1DK4); AF2372_Afu – Archeoglobus fulgidus IMPase/FBPase AF2372 (1LBV); TM1415_Tma – Thermotoga maritima IMPase TM1415 (2P3N); PIPase_Rno – Rattus norvegicus 3'-phosphoadenosine 5'-phosphate and inositol 1,4-bisphosphate phosphatase (1JP4). Secondary structure elements above the sequence refer to the crystal structure of M. tuberculosis SuhB. [file 1472-6807-7-55-S2.png]
