# Supplementary material for: Dimerization of inositol monophosphatase Mycobacterium tuberculosis SuhB is not constitutive, but induced by binding of the activator Mg2+
Source: BMC Struct Biol. 2007 Aug 28;7:55. doi: 10.1186/1472-6807-7-55 (PMC2080633; doi:10.1186/1472-6807-7-55)
Supplement: Additional file 3 — References for Table 2. This file contains references for PDB entries cited in Table 2 of the main text. [file 1472-6807-7-55-S3.pdf]

Dimerization of inositol monophosphatase *Mycobacterium tuberculosis* SuhB is not constitutive, but induced by binding of the activator Mg^{2+}

Alistair K. Brown[1]*, Guoyu Meng[1,4]*, Hemza Ghadbane[1]*, David J. Scott[2], Lynn G. Dover[1], Jérôme Nigou[3], Gurdyal S. Besra[1§] and Klaus Fütterer[1]

Additional file 3: Supplementary Information

References for PDB entries cited in Table 2 of the main text.

1IMA - [1]; 2CZH - [2]; 2BJI - [3]; 1FBP - [4]; 1DCU - [5]; 1LBV - [6]; 1DK4 - [7]; 1QGX - [8]; 1INP - [9]; 1JP4 - [10]

1. Bone R, Frank L, Springer JP, Pollack SJ, Osborne SA, Atack JR, Knowles MR, McAllister G, Ragan CI, Broughton HB, et a: **Structural analysis of inositol monophosphatase complexes with substrates.** *Biochemistry* 1994, **33**:9460-9467.
2. Arai R, Ito K, Ohnishi T, Ohba H, Akasaka R, Bessho Y, Hanawa-Suetsugu K, Yoshikawa T, Shirouzu M, Yokoyama S: **Crystal structure of human myo-inositol monophosphatase 2, the product of the putative susceptibility gene for bipolar disorder, schizophrenia, and febrile seizures.** *Proteins* 2007, **67**:732-742.
3. Gill R, Mohammed F, Badyal R, Coates L, Erskine P, Thompson D, Cooper J, Gore M, Wood S: **High-resolution structure of myo-inositol monophosphatase, the putative target of lithium therapy.** *Acta Crystallogr D Biol Crystallogr* 2005, **61**:545-555.
4. Liang JY, Huang S, Zhang Y, Ke H, Lipscomb WN: **Crystal structure of the neutral form of fructose 1,6-bisphosphatase complexed with regulatory inhibitor fructose 2,6-bisphosphate at 2.6-A resolution.** *Proc Natl Acad Sci U S A* 1992, **89**:2404-2408.
5. Chiadmi M, Navaza A, Miginiac-Maslow M, Jacquot JP, Cherfils J: **Redox signalling in the chloroplast: structure of oxidized pea fructose-1,6-bisphosphate phosphatase.** *EMBO J* 1999, **18**:6809-6815.
6. Stieglitz KA, Johnson KA, Yang H, Roberts MF, Seaton BA, Head JF, Stec B: **Crystal structure of a dual activity IMPase/FBPase (AF2372) from Archaeoglobus fulgidus. The story of a mobile loop.** *J Biol Chem* 2002, **277**:22863-22874.
7. Stec B, Yang H, Johnson KA, Chen L, Roberts MF: **MJ0109 is an enzyme that is both an inositol monophosphatase and the 'missing' archaeal fructose-1,6-bisphosphatase.** *Nat Struct Biol* 2000, **7**:1046-1050.
8. Albert A, Yenush L, Gil-Mascarell MR, Rodriguez PL, Patel S, Martinez-Ripoll M, Blundell TL, Serrano R: **X-ray structure of yeast Hal2p, a major target of lithium and sodium toxicity, and identification of framework interactions determining cation sensitivity.** *J Mol Biol* 2000, **295**:927-938.
9. York JD, Ponder JW, Chen ZW, Mathews FS, Majerus PW: **Crystal structure of inositol polyphosphate 1-phosphatase at 2.3-A resolution.** *Biochemistry* 1994, **33**:13164-13171.
10. Patel S, Yenush L, Rodriguez PL, Serrano R, Blundell TL: **Crystal structure of an enzyme displaying both inositol-polyphosphate-1-phosphatase and 3'-phosphoadenosine-5'-phosphate phosphatase activities: a novel target of lithium therapy.** *J Mol Biol* 2002, **315**:677-685.
